# Destabilization of EpCAM dimer is associated with increased susceptibility towards cleavage by TACE

**DOI:** 10.7717/peerj.11484

**Published:** 2021-05-21

**Authors:** Tomaž Žagar, Miha Pavšič, Aljaž Gaber

**Affiliations:** Department of Chemistry and Biochemistry, Faculty of Chemistry and Chemical Technology, University of Ljubljana, Ljubljana, Slovenia

**Keywords:** EpCAM, Dimerization, TACE, Proteolytic cleavage

## Abstract

The cell-surface protein EpCAM is a carcinoma marker utilized in diagnostics and prognostics, and a promising therapeutic target. It is involved in nuclear signaling via regulated intramembrane proteolysis (RIP). Many aspects of this process are not fully understood, including the events at the molecular level leading to the exposure of cleavage sites, buried at the dimerization interface. To investigate the effect of dimer stability on cleavage susceptibility we prepared two mutants of human EpCAM ectodomain: a monomeric form, and a disulfide-stabilized dimeric form. We show that the disulfide-stabilized dimer is resistant to tumor necrosis factor-α-converting enzyme (TACE) cleavage, while the monomeric form is more susceptible than the predominantly dimeric wild type. This provides experimental evidence that the oligomeric state of EpCAM is a determinant in RIP and demonstrates the usefulness of the oligomeric state-specific mutants in investigations of EpCAM biological function.

## Introduction

Epithelial cell adhesion molecule (EpCAM, CD326) is a homodimeric type I cell-surface glycoprotein, frequently overexpressed in epithelial cancers (Went et al., 2004). EpCAM overexpression in carcinomas is linked to cancer proliferation, migration, and metastasis (Trzpis et al., 2007), and is associated with poor prognosis (Spizzo et al., 2002, 2004; Varga et al., 2004; Fong et al., 2007; Brunner et al., 2008; Scheunemann et al., 2008; Chen et al., 2014). This makes it a promising target for cancer diagnostics and treatment (reviewed in Simon et al., 2013).

EpCAM exerts its biological function as a regulator of epithelial cell-cell adhesion (Gires et al., 2020) and is involved in signaling via two mechanisms—regulated intramembrane proteolysis (RIP) (Maetzel et al., 2009) and interaction with epithelial growth factor receptor (EGFR) (Pan et al., 2018; Liang et al., 2018). In RIP, EpCAM is first cleaved by α-secretase ADAM10 or ADAM17, also known as tumor necrosis factor-α-converting enzyme (TACE), within its extracellular part (EpEX), which leads to the release of the almost complete ectodomain, except for a short 2,4 kDa part that remains tethered to the membrane. Next, EpCAM is processed by the γ-secretase complex, resulting in a soluble short intracellular tail (EpIC). EpIC then interacts with proteins of β-catenin/Wnt-signaling pathway to form the EpIC-FHL2-Lef1-β-catenin complex (Maetzel et al., 2009; Tsaktanis et al., 2015), which in turn regulates expression of oncogenes (Maetzel et al., 2009; Lu et al., 2010; Huang et al., 2011; Chaves-Pérez et al., 2013; Yu, Ma & Wang, 2017).

The quaternary structure of EpCAM appears to be inherently linked to its biological function. Initial models predicted various oligomeric states; however, later it has been demonstrated that the ectodomain forms a stable dimer (Pavšič et al., 2014; Gaber et al., 2018). This dimer is also representative of the full-length molecule where the transmembrane helix dimerization could further contribute to the dimer stability (Pavšič et al., 2014; Gaber et al., 2018). Mapping of the TACE cleavage sites onto the EpEX dimerization surface (Tsaktanis et al., 2015) indicated that the cleavage by TACE can take place only when EpCAM is in a monomeric state. Molecular docking of the TACE catalytic domain on EpEX (Gaber, Lenarčič & Pavšič, 2020) also supports this hypothesis by demonstrating that EpCAM dimerization sterically hinders access of TACE to the cleavage site. However, the effect of EpCAM oligomeric state on susceptibility to TACE cleavage has never been experimentally addressed, partly due to the inability to manipulate the EpCAM/EpEX oligomeric state.

Here, we present two designed EpEX mutants with distinct oligomeric states. First, by analysis of EpEX dimerization surface as found in crystal structure and further explored by molecular dynamics simulations, we devised a set of three single-site mutations that prevent EpEX dimerization and therefore result in persistently monomeric EpEX. Second, we generated a covalently stabilized EpEX dimer by introducing a cysteine residue at the C-terminal tail of EpEX, leading to the formation of a disulfide bridge between the two subunits. Third, we demonstrate that these two mutant proteins can be employed to study the effect of EpEX’s oligomeric state on its interactions with other proteins. By comparing the extent of their proteolytic degradation with TACE’s ectodomain (TACE-EX) in a solution we provide experimental evidence that EpCAM can only be efficiently cleaved as a monomer, strongly indicating the importance of the role of EpCAM’s oligomerization in the regulation of RIP.

## Materials & Methods

### In silico construction and molecular dynamics simulations of EpEX

As input structures EpEX dimer from the crystal structure (PDB ID 4mzv) (Pavšič et al., 2014) and its in silico mutated TYloop^mut^ form (K83D, P84D, L88D) were used. The mutations were introduced using UCSF Chimera (Pettersen et al., 2004). First, topology files were prepared using VMD 1.9.3 with the psfgen plugin (http://www.ks.uiuc.edu/Research/vmd/) (Humphrey, Dalke & Schulten, 1996). Briefly, protein molecules were solvated (20 Å margin on each side, giving a box of approximately 100 × 100 × 100 Å) and the system neutralized by the introduction of sodium or chloride ions. Next, all-atom molecular dynamics runs were performed using NAMD (http://www.ks.uiuc.edu/Research/namd/) 2.11 (Phillips et al., 2005) under periodic boundary conditions and using full-system periodic electrostatics. To prevent the introduction of artefacts, the protein atoms were kept at a fixed position during the initial minimization step, and only the water molecules and ions were allowed to move freely. Following this, restrictions were removed and the system was equilibrated at a constant pressure of 1 atm using Langevin piston, and a constant temperature of 310 K using Langevin dynamics for 5 ns with a timestep of 2 fs. Trajectory files were analyzed using UCSF Chimera (Pettersen et al., 2004) to obtain inter-residue distance *vs*. time. To obtain the inter-residue contact network we used USCF Chimera in conjunction with Cytoscape 3.8.2 (Shannon et al., 2003) and StructureViz2 plugin (Morris et al., 2007).

### Protein cloning, expression and purification

Both EpEX mutants—TYloop^mut^ (K83D, P84D and L88D) and L264C—were derived from EpEX (residues 24-265) with mutated N-glycosylation sites (N74Q, N111Q and N198Q) (Pavšič et al., 2014). The final K265 is followed by residues LE, translated from *Xho*I scar, and a His_6_-tag. The wild-type signal sequence was replaced with the honeybee signal sequence (MKFLVNVALVFMVVYISYIYA) to increase expression yields (Pavšič et al., 2014) in insect cells. Protein coding sequences were cloned in pFastBac1. Recombinant bacmids were prepared in DH10MultiBac (Berger, Fitzgerald & Richmond, 2004) according to the Bac-to-Bac system (Invitrogen) and transfected into insect cells *Spodoptera frugiperda* Sf9 (Thermo Fisher Scientific, Waltham, MA, USA) using Turbofect according to manufacturer’s instructions (Thermo Fisher Scientific, Waltham, MA, USA). Baculoviral stock solution (V0) was amplified twice, each time for three days, to ensure high viral titer for efficient expression of recombinant proteins.

All proteins were expressed in insect cell line *Spodoptera frugiperda* Sf9 (Thermo Fisher Scientific, Waltham, MA, USA) and purified as described before (Pavšič et al., 2014). Harvesting was done three days post-infection with V2 viral stock, and the cell culture supernatant was obtained by centrifugation (10 min at 10,000 × *g* at 4 °C). The pH of the cleared supernatant was adjusted to 8.0 by adding Tris-HCl to a final concentration of 2.5 mM during mixing, and protease inhibitor phenylmethylsulfonyl fluoride (PMSF) was added to a final concentration of 1 mM. The solution was centrifuged for the second time (10 min at 10,000 × *g* at 4 °C) and applied to a 5 ml cOmplete His-Tag Purification Column (Roche, Switzerland), previously equilibrated with 20 mM sodium phosphate buffer, pH 7.5, 500 mM NaCl. Recombinant proteins were eluted using an imidazole gradient (final concentration of 500 mM) and dialyzed overnight against 20 mM HEPES, pH 8 at 4 °C. The purification then proceeded with an ion-exchange chromatography on 5 ml HiTrap Q HP (GE Healthcare, Chicago, IL, USA), equilibrated with 20 mM HEPES, pH 8. Proteins were eluted using NaCl gradient (final concentration of 500 mM). Fractions containing recombinant proteins of interest (as determined by SDS-PAGE) were pooled and applied to size exclusion column HiLoad^™^ 16/600 (GE Healthcare, Chicago, IL, USA), equilibrated with 20 mM HEPES, pH 8, 100 mM NaCl. Pooled fractions were concentrated and stored at −80 °C.

Recombinant baculoviruses harboring fragment coding for TACE-EX (residues 1–824, with N-terminal honeybee melittin signaling sequence and C-terminal His_6_-tag) were prepared in the same way as the EpEX mutants. Also, the expression and purification procedure were the same, except for the addition of Halt^™^ Protease and Phosphatase Inhibitor Single-Use Cocktail, EDTA-Free (Thermo Fisher Scientific, USA) after the dialysis step.

### Molecular weight analysis

Molecular weights (MWs) of the proteins were assessed by Size exclusion chromatography coupled with right- and low-angle laser light scattering detectors (SEC-RALLS/LALLS system) OMNISEC RESOLVE+REVEAL (Malvern Panalytical, UK) using a Superdex^®^ 200 Increase 10/300 GL column (GE Healthcare/Cytiva, USA) for protein separation.

A total of 150 μg of each protein was injected per run and each protein sample was analyzed in a duplicate. The system operated at 25 °C and a flow rate of 0.5 ml/min. For all samples, the same buffer was used namely, 20 mM HEPES, pH 7.5, 100 mM NaCl. BSA (Thermo Fischer Scientific, Waltham, MA, USA) was used as a calibration standard. Retention volumes were adjusted according to BSA peak position. The MW distributions were calculated using the software OMNISEC version 11.20 (Malvern Panalytical, UK).

### CD analysis

Circular dichroism spectra were recorded on a J-1500 CD spectrometer (Jasco, Easton, MD, USA) in a 1 mm quartz cuvette (Hellma, Germany) at 25 °C. Spectra were registered in the wavelength range between 200 and 250 nm with scanning speed set to 20 nm/min and the bandwidth and data pitch to 1 nm. Four scans per measured sample were averaged and corrected for the sample buffer (20 mM sodium phosphate, pH 7.4). Protein concentration was 0.25 mg/ml.

### Proteolytic cleavage of EpEX and its mutants

Proteins were dialyzed against the reaction buffer 20 mM sodium phosphate, pH 7.4, 10 μM ZnCl_2_, complemented with cOmplete^™^ Mini EDTA-free Protease Inhibitor Cocktail (Roche, Switzerland), using a Slide-A-Lyzer^®^ MINI Dialysis device (10K MWCO membrane, 10–100 ul) (Thermo Fischer Scientific, Waltham, MA, USA), according to manufacturer’s instruction.

For a single cleavage experiment, 15 µg of EpEX or its mutant were mixed with 7.5 µg of TACE-EX in a final volume of 30 µl and incubated for 0, 2, or 6 h at 37 °C with mixing at 300 RPM. The reaction was stopped by the addition of 4 × SDS loading buffer containing DTT, and subsequent incubation at 95 °C for 5 min. The control samples without the TACE-EX were incubated for 6 h, while the initial samples were not incubated at 37 °C.

For the Western blot analysis, one-tenth of the reaction sample was separated using SDS-PAGE. Separated proteins were transferred onto Immobilon^®^–FL PVDF (Merck Millipore, Burlington, MA, USA) membrane using wet transfer system Mini Trans-Blot^®^ (Bio-Rad, Irvine, CA, USA). Blocking was done using fluorescent blocking solution Immobilon^®^ Block—FL (Merck Millipore, Burlington, MA, USA) supplemented with 0.2% Tween 20 for 1 h at room temperature during constant mixing. Mouse anti-His Tag antibodies (ABIN195462; Antibodies-Online, Germany) were diluted 1:1,000 directly into the blocking solution and the membrane was incubated for an additional hour. After three 5-min washes with TBST (0.2% Tween 20), the membrane was incubated in goat anti-mouse IgG (H+L) superclonal recombinant secondary antibodies conjugated with Alexa Fluor 647 dye (Thermo Fischer Scientific, Waltham, MA, USA) diluted 1:5,000 in blocking solution supplemented with 0.2% Tween 20 at room temperature for 1 h and washed six times for 5 min with TBST. Detection was performed using ChemiDoc^™^ MP imaging system (Bio-Rad, Irvine, CA, USA) with a preset program for imaging AlexaFluor 647 dye. Quantification was done using software ImageLab version 6.0.1 (Bio-Rad, Irvine, CA, USA).

The band intensities of three different blots were first scaled according to the sum of the signal of the initial samples. For each protein, the average of scaled control samples incubated without the TACE-EX was used for normalization.

### Statistics

Results of proteolytic cleavage experiments are represented as a mean value ± SEM. One-way ANOVA (*p* < 0.001). Analyses were done with GraphPad Prism version 8.

## Results

### Rational design of monomeric and dimeric mutants

**Figure 1:**
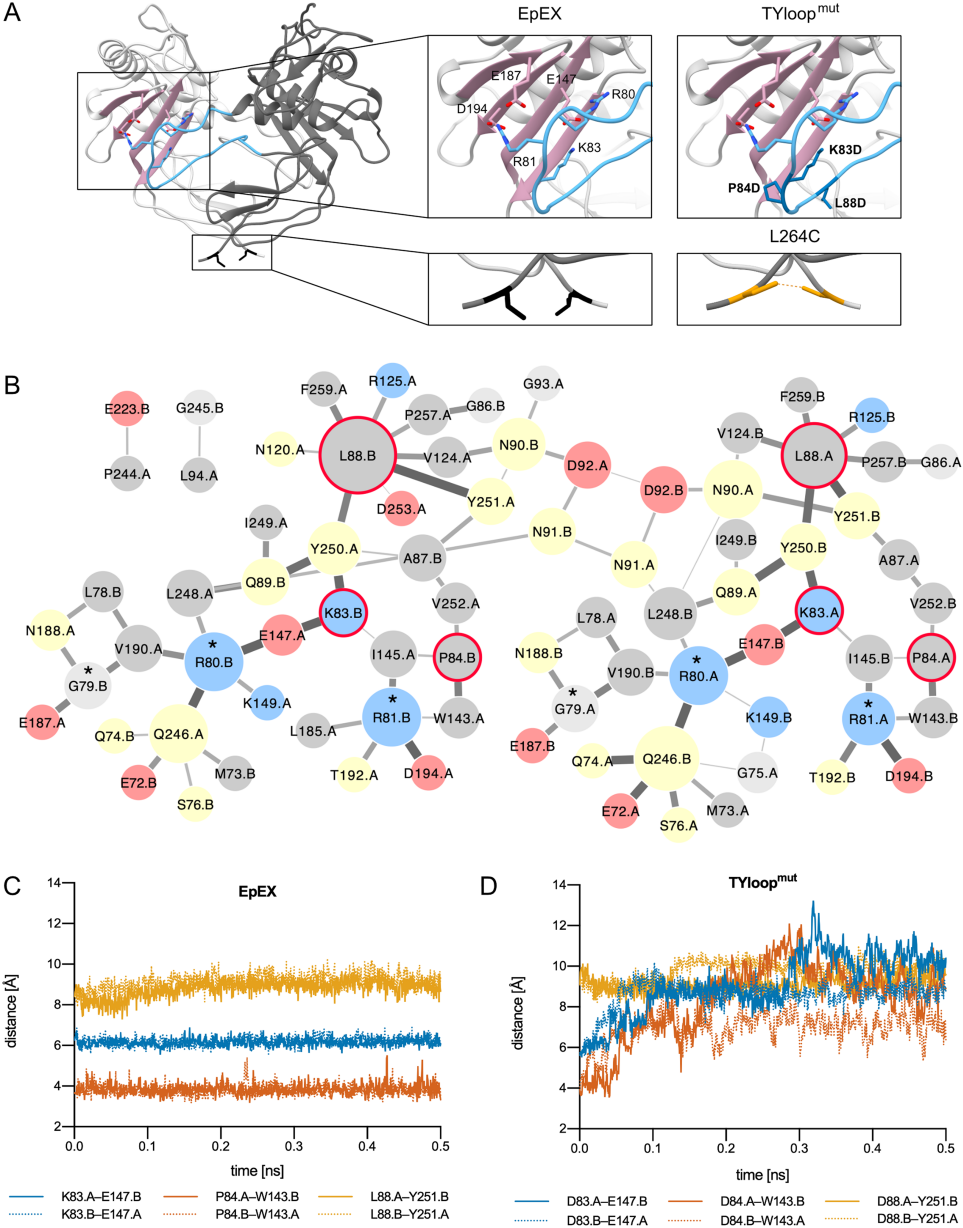
EpEX dimer and the designed mutants. (A) The structure of EpEX dimer and the designed mutants. The dimer subunits are colored with different shades of grey. The β-sheet of the CD is depicted in pink and the TYloop is depicted in blue. The sidechains of key interacting residues, as inferred from the crystal structure, are shown as sticks. Residues that were mutated in TYloop^mut^ and L264C are colored dark blue and orange, respectively. (B) Frequency of inter-subunit inter-residue contacts during EpEX MD simulation. The width of the line between two residues correlates with the frequency of contact formation—a wider line represents more frequent interaction. The size of circles corresponds to the number of interactions with other residues—a larger circle represents more interactions. Residues are colored according to their charge and polarity: positively charged blue, negatively charged red, polar yellow and non-polar grey. The residues of protease-sensitive site Gly79-Arg80-Arg81 are annotated with a star (*). Residues that were mutated in TYloop^mut^ are encircled with a red line. (C) Changes in the key inter-subunit inter-residue distances during the first 0.5 ns of the wild type MD simulation. (D) Changes in the key inter-subunit inter-residue distances during the first 0.5 ns of the mutant TYloop^mut^ MD simulation. For distance calculations in C and D, equivalent atoms were used for both wild type and mutated residues (C_γ_ for K/D83, P/D84 and L/D88); for E148 C_δ_ was used, and for W143 and Y251 C_ζ_ was used.

We devised mutations that either additionally stabilize or destabilize EpEX dimer. For this, the crystal structure of EpCAM ectodomain dimer was used as the representation of the native state (Pavšič et al., 2014). In this dimer, the long loop of the thyroglobulin domain (TYloop) forms extensive interactions with the β-sheet in the C-terminal domain (CD) of the juxtaposed subunit. Here, electrostatic interactions between Arg80, Arg81, and Lys83 (all within the TYloop), and a negatively charged patch formed by Glu147, Glu187, Asp194 (all within β-sheet) appear to be critical for dimer stabilization (Pavšič et al., 2014) (Fig. 1A). In the design process three additional aspects were considered: (1) mutations within TYloop, which is in monomeric species completely exposed to solvent and probably structurally disordered, most probably wouldn’t interfere with subunit folding; (2) mutations introduced within the β-sheet of the C-terminal domain could interfere with domain folding; (3) protease-sensitive site Gly79-Arg80-Arg81 (Thampoe, Ng & Lloyd, 1988; Perez & Walker, 1989; Schön et al., 1993) has to be retained so that the mutant protein can potentially be used in experiments addressing processing at this site.

The mutant with impaired dimerization propensity was designed by introducing negatively charged residues into the TYloop, which would result in electrostatic repulsion between this region and the negatively charged patch within the β-sheet of the juxtaposed subunit. We prepared in silico mutants where residues in the range 83–88 were changed one-by-one to aspartates using UCSF Chimera (Pettersen et al., 2004; Goddard, Huang & Ferrin, 2007); for each mutation, the most probable rotamer from the Dunbar library was chosen (Shapovalov & Dunbrack, 2011). The effect of mutations on dimer stability was evaluated using the ‘Protein interfaces, surfaces and assemblies’ service PISA at the European Bioinformatics Institute (Krissinel & Henrick, 2007). The highest impact on solvation free energy gain upon the formation of the dimer interaction interface was achieved by a set of three mutations K83D, P84D and L88D, and this mutant was named TYloop^mut^ (Fig. 1A, Table S1).

To get detailed insight into the dimer-stabilizing interactions and their time distribution we performed an all-atom 5 ns molecular dynamics (MD) simulation and analyzed the inter-residue contacts between the two subunits of the native-like EpEX dimer in terms of their frequency (Fig. 1B). We observed several frequent ionic interactions (salt bridges) as already inferred from the static crystal structure; however, the MD simulation revealed some additional prominent residue clusters, particularly those with Leu88 in the center, and a small cluster involving Pro84. This is in good agreement with the dimer interface analysis described above. To evaluate the effect of the designed mutations *in silico*, we performed an analogous MD simulation run also with the mutated EpEX form—the EpEX TYloop^mut^. Trajectory analysis of inter-residue distance *vs*. time for selected residue pairs (K83–E147, P84—W143, L88—Y251 for wild type, and D83–E147, D84—W143, D88—Y251 for the EpEX TYloop^mut^) revealed that the distance *vs*. time is very stable for the wild type form (Fig. 1C). On the contrary, the distance plot for the equivalent atom pairs shows a considerable jump to higher values indicating the start of dimer dissociation (Fig. 1D), except for the residue pair involving Y251 where the distance in the wild type form is already larger. This further supports the proposed destabilizing effect of designed mutations of the EpEX TYloop^mut^ form.

A covalently stabilized dimer mutant was prepared by introducing a cysteine residue (L264C) near the C-terminus of the EpEX (Fig. 1A). The formation of an EpEX dimer via native interactions would bring the two cysteine residues from juxtaposed subunits into close proximity thus enabling the formation of a disulfide bond, thereby covalently linking the subunits and preventing their dissociation. The oxidative potential of the environment where the mutated site is located in the expressed protein—the lumen of the secretory pathway compartments or the extracellular space—would contribute to the probability that a stable disulfide bond is indeed formed. The C-terminus of EpEX was shown to be flexible and the mutation spot is distant from the major well-structured part of the subunit; therefore, the mutation L264C was not predicted to introduce significant structural perturbations (Gaber et al., 2018).

A mutant non-glycosylated EpEX variant was used as starting template for both described mutants to avoid the effect of heterogeneous and non-native glycosylation due to expression in insect cells. It had already been demonstrated that this variant forms stable dimers (Pavšič et al., 2014; Gaber et al., 2018).

### The designed EpEX mutants occupy distinct oligomeric states

**Figure 2:**
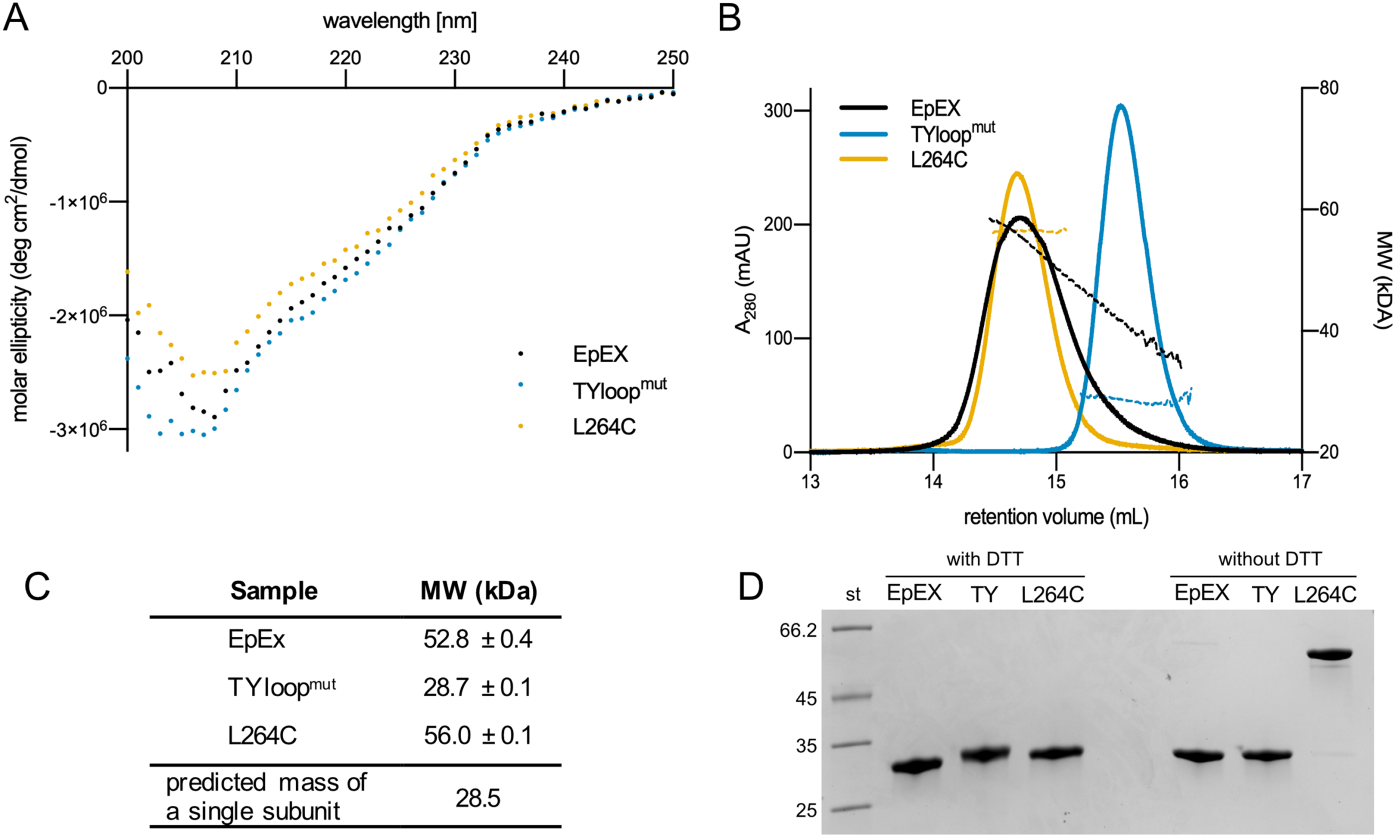
Quality control and oligomeric state analyses of purified EpEX, TYloop^mut^ and L264C. (A) Overlay of CD spectra. (B) An overlay of representative SEC elution spectra of purified proteins with the corresponding MW fits. (C) The summary of molecular weights, determined with SEC-RALS/LALS. For each sample, mean values and s.d. from two injections are presented. The predicted mass of a single subunit was calculated from EpEX amino-acid sequence with the ProtParam tool (Gasteiger et al., 2005). (D) SDS-PAGE analysis under reducing (with DTT) and non-reducing (w/o DTT) conditions.

EpEX with native-like dimerization propensity and both designed mutant EpEX variants (TYloop^mut^ and L264C) were expressed in insect cells and purified using a series of chromatographic runs. Final yields of designed mutants were lower than that of EpEX—approx. 2 mg of purified protein per liter of cell culture, compared to 4–5 mg for EpEX; however, they were sufficient for further analysis. The CD spectra of all three purified proteins are very similar (Fig. 2A) which indicates that the introduced mutations did not significantly affect the EpEX structure and that both TYloop^mut^ and L264C are properly folded.

We investigated the oligomeric state of EpEX, TYloop^mut^ and L264C using size exclusion chromatography (SEC), MW determination via light scattering, and SDS-PAGE. First, in SEC elution chromatograms all variants gave a single peak (Fig. 2B). This indicates that there are no significant aggregates in any of the samples and that there is one predominant oligomeric species present in each of the samples. The peaks relative to the TYloop^mut^ and L264C are symmetric, while the EpEX peak exhibits slight tailing which could be attributed to the small amount of monomeric species. The position of the peak for EpEX and L264C overlap, suggesting that their oligomeric state is the same—a dimer, which has already been demonstrated for EpEX (Pavšič & Lenarčič, 2011; Pavšič et al., 2014; Gaber et al., 2018).TYloop^mut^ elutes at higher elution volume than EpEX and L264C indicating a smaller hydrodynamic radius, indicating a different oligomeric state than for EpEX and L264C.

Next, we used SEC-RALLS/LALLS to determine the absolute MW of the species present in the solution (Fig. 2C). The determined MW of the species present in the main part of the EpEX peak is 52.8 ± 0.4 kDa, while for the tailing part it decreases to 30 kDa. These two extremes roughly correspond to the dimer and monomer with calculated MW of 54.4 and 28.2 kDa, respectively, confirming the equilibrium between the two species. On the other hand, the plot of MW vs. elution volume for the two mutants indicates that each mutant is present in the solution as a species with a single oligomeric state. The determined MW for TYloop^mut^ of 28.7 ± 0.1 kDa is in good agreement with the monomeric form, while the MW for L264C (56.0 ± 0.1 kDa) indicates a dimeric form. The latter, together with no observed peak tailing, also confirms the expectation that the disulfide bond formed between the introduced cysteine residue from juxtaposed dimer subunits would prevent the dimer dissociation.

Last, we used SDS-PAGE under reducing and non-reducing conditions to analyze whether the disulfide linkage between the subunits of the L264C dimer indeed formed as designed (Fig. 2D). Under reducing conditions, EpEX and both mutants have the same electrophoretic mobility, and the apparent MW corresponds to that calculated from the amino acid sequence (28 kDa). However, under non-reducing conditions, the electrophoretic mobility of the L264C mutant is significantly lower and corresponds to a dimer (56 kDa), in contrast to the EpEX and TYloop^mut^ where electrophoretic mobility is unchanged. Only a very small amount of L264C mutant is monomeric under reducing conditions, which clearly shows that the majority of subunits in L264C are covalently linked together in pairs by a disulfide bond that results from the L264C mutation.

Based on SEC elution profiles, determined MW and SDS-PAGE analysis we conclude that we have successfully prepared two EpEX mutants with distinct oligomeric states. As neither of them exhibits equilibrium between the two monomeric and dimeric states, they are suitable candidates for further investigations of how the oligomeric state of EpEX affects EpCAM’s role in cellular processes such as RIP-mediated signaling.

### EpEX monomeric mutant is more prone to degradation by TACE

Mapping of TACE cleavage sites to EpEX dimer (Tsaktanis et al., 2015) indicated that the oligomeric state of EpCAM could play a role in the regulation of RIP. Namely, the cleavage sites appear to be accessible only if the EpCAM is monomeric. To address this experimentally we investigated the susceptibility of EpEX, plus the TYloop^mut^ (monomer) and L264C (dimer) mutants towards cleavage by TACE ectodomain (TACE-EX) in vitro.

**Figure 3:**
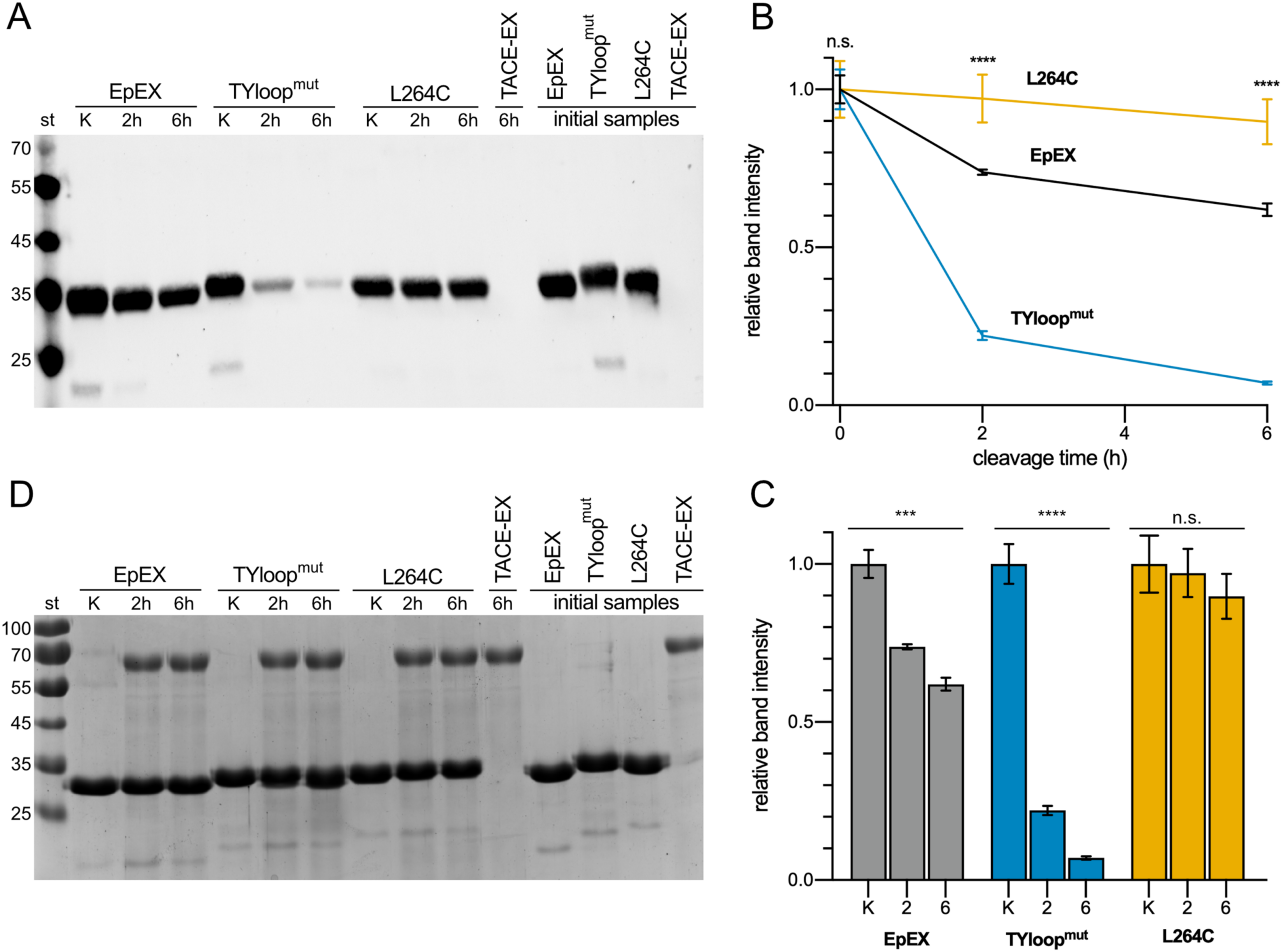
Cleavage of EpEX and its mutants by TACE-EX. (A) A representative Western blot analysis with anti-histidine tag antibodies. The control samples without the TACE-EX and the control for TACE-EX alone were incubated for 6 h. (B) The comparison of time-dependent reduction of signal intensity. The control sample that was incubated without the TACE-EX is given at 0 h. (C) Histograms representing the reduction of the signal intensity for each analyzed protein. (D) SDS-PAGE analysis of the same representative Western blot analysis. A one-way ANOVA test was performed to identify statistically significant differences between the mean values (*n* = 3): n.s. non-significant differences, ***p < 0.0002, ****p < 0.0001.

The cleavage extent was estimated via quantification of the protein remaining uncleaved, which was detected using anti-His_6_-tag antibodies targeting the His_6_-tag present in all three EpEX variants. As the tag gets released after the cleavage, the extent of cleavage can be evaluated by measuring the reduction of the signal (Fig. 3A). This approach was selected because the cleavage site by TACE is relatively close to the C-terminus of EpEX, and the cleavage results in the liberation of a short fragment (2.1 kDa) which is challenging to be detected and accurately quantified using SDS-PAGE. At the same time, the difference in MW between non-cleaved and cleaved is EpEX too small; therefore, the cleavage product is not easily resolvable from the uncleaved EpEX.

Our results show that roughly a quarter of the EpEX was cleaved after 2 h, and almost 40 % after 6 h (Fig. 3B). In contrast to this, only insignificant cleavage of L264C was observed (Figs. 3B, 3C). This confirms the hypothesis that cleavage by TACE-EX is inefficient when EpCAM is a dimer. On the other hand, the cleavage of TYloop^mut^ is significantly more efficient compared to EpEX at both time points, as almost more than 75% was processed after 2 h and more than 90% after 6 h, further confirming that the oligomeric state is implicated in the regulation of RIP. At the same time, this demonstrates that our designed mutants are indeed useful to study the role of EpCAM oligomerization in RIP. Parallel SDS-PAGE analysis (Fig. 3D) of the same samples that were analyzed by Western blotting shows that the band intensity corresponding to all three EpEX variants is the same at each time point which indicates that the His_6_-tag signal of cleaved protein indeed diminishes due to cleavage near the C-terminus.

Surprisingly, the signal corresponding to TACE-EX on our Western blots could only be observed after a prolonged exposition. Since the TACE-EX bears a C-terminal His_6_-tag—similarly to the EpEX variants—a stronger signal would be expected on a Western blot after detection using anti-His_6_-antibody (Fig. 3A). However, on SDS-PAGE the band corresponding to TACE-EX is visible (Fig. 3D). The apparent MW is larger than that calculated from the amino acid sequence (52 kDa), and the difference could be attributed to glycosylation at one or more of the nine N-glycosylation sites present in TACE-EX. We speculate that the reduced signal intensity on Western blots is a result of a C-terminal truncation, which occurred during the purification, resulting in the loss of His_6_-tag. Similar observations of the loss of C-terminal tags after purifications were also observed by others for both TACE (Johannes, Bercher & Blobel, 2000) and its homologue ADAM10 (Brummer et al., 2018). They were attributed to C-terminal autoproteolytic processing (Johannes, Bercher & Blobel, 2000; Brummer et al., 2018).

We believe that the observed cleavage extent is indeed linked to the EpEX oligomeric state and activity of TACE-EX. First, all cleavage reaction mixtures contained a cocktail of inhibitors targeting proteases of all other classes. Next, all proteins were isolated using the same procedure and would thus have the same contaminants. However, when these proteins are incubated without TACE-EX (Figs. 3A, 3D), no degradation is observed.

## Discussion

Homo-oligomerization has always been recognized as an important aspect of EpCAM function. Initial observations of its role in cell-cell adhesion were attributed to its ability to form cell-cell contacts through the self-assembly of EpCAM molecules from adjacent cells (Balzar et al., 2001; Trebak et al., 2001). A recent investigation of EpCAM oligomerization did not find any direct evidence of higher-order oligomerization, and it also confirmed speculations that EpCAM exists predominately as a cis-dimer—a dimer formed by the association of two subunits from the same cell (Gaber et al., 2018). However, differences between monomeric and dimeric forms of EpCAM and their specific functional roles were never investigated. This was largely due to the lack of options for confining EpCAM to either a monomeric or dimeric state without considerably changing experimental conditions, for example by lowering the pH (Pavšič & Lenarčič, 2011). To make such investigations possible, we prepared both monomeric as well as an obligate-dimeric mutant of EpCAM soluble ectodomain.

We showed that three mutations in EpEX TY-loop (K83D, P84D and L88D) successfully prevent dimerization. This confirms conclusions based on EpEX crystal structure (Pavšič et al., 2014) that interactions between TY-loop and β-sheet are essential for dimer formation. Even at a concentration of 1.5 mg/ml (~50 µM), the mutant TYloop^mut^ showed no signs of dimerization (Figs. 2B, 2C). The wild type-like EpEX is almost exclusively dimeric at this concentration (Figs. 2B, 2C), which is also in agreement with previously published small-angle X-ray scattering (SAXS) data, collected at this concentration (Gaber et al., 2018). While the wild type-like EpEX exists both as monomer and dimer (in equilibrium), the L264C mutant is constitutively dimeric (Figs. 2B, 2C), due to disulfide covalent linkage between the two subunits (Fig. 2D).

Using the prepared mutants, we also experimentally in vitro confirmed the role of EpCAM oligomeric state in RIP, specifically the first cleavage by TACE, for the first time. The lack of any significant TACE-EX cleavage of L264C mutant and the almost complete cleavage of TYloop^mut^ mutant (Fig. 3) clearly show that EpCAM needs to be monomeric for the cleavage to take place. The wild-type-like EpEX, which was shown to be a stable dimer in solution, was also cleaved, although to a lesser extent than the monomeric mutant. We hypothesize that TACE-EX readily cleaves monomers as soon as they become available due to the dissociation of subunits in a dimer. However, to evaluate the potential TACE innate ability to promote EpEX dissociation, experiments in cellula are needed. For example, an amphipathic α-helical region at the C-terminal part of TACE-EX called Conserved Adam seventeeN Dynamic Interaction Sequence (CANDIS) was shown to be essential for substrate recognition in cleavage of type I transmembrane proteins such as IL-R6 (Düsterhöft et al., 2014). Whether interaction with CANDIS is also important for EpCAM cleavage and potentially even its oligomeric state is still to be discovered. Since CANDIS also interacts with the cell membrane (Düsterhöft et al., 2015) such experiments require the use of full-length membrane-embedded proteins to adequately represent the native conditions.

**Figure 4:**
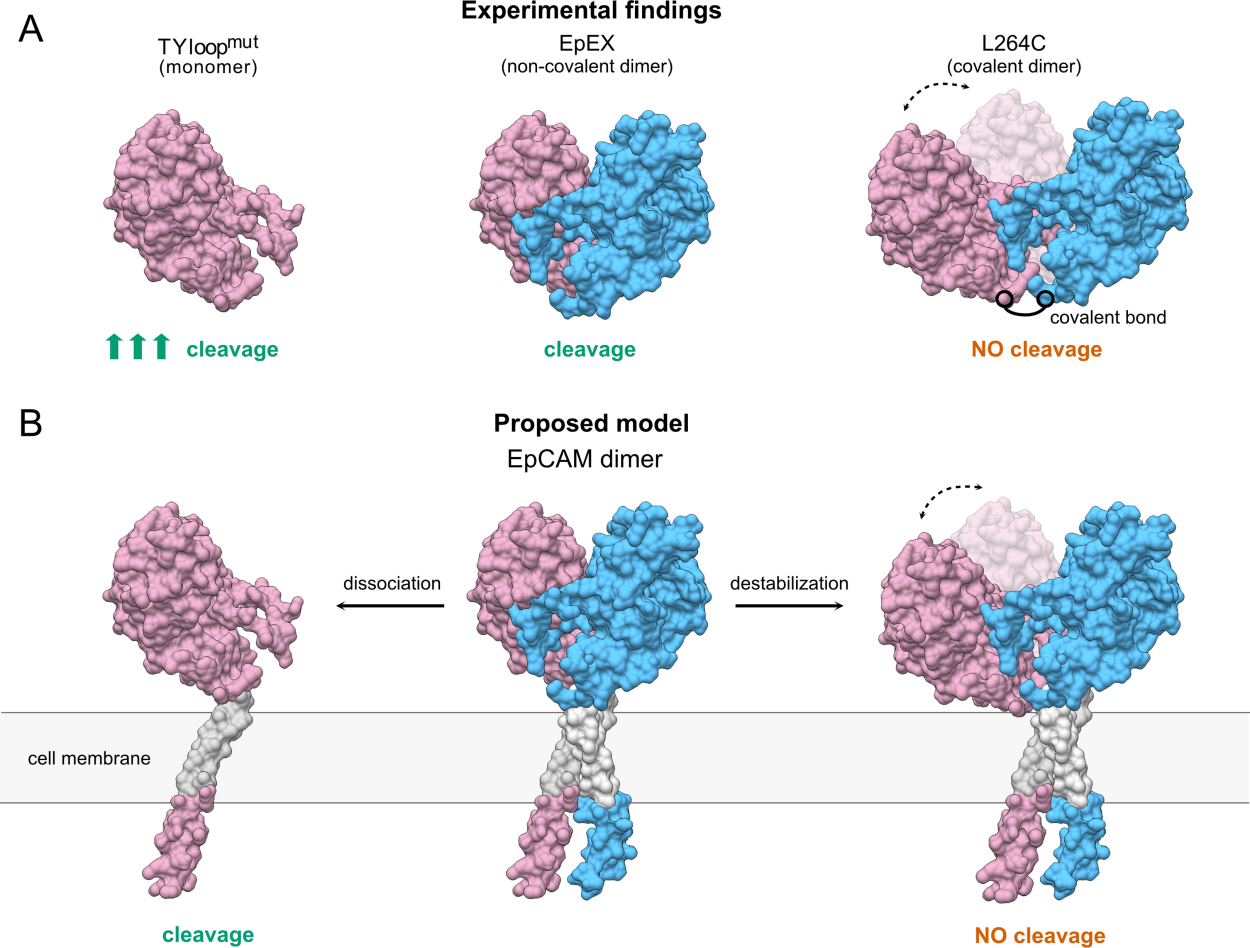
The proposed model of TACE-mediated EpCAM proteolytic processing, based on EpEX cleavage data. (A) A Summary of experimental findings. The increased cleavage of the TYloop^mut^ compared to EpEX is indicated by three arrows. (B) The proposed model of EpCAM cleavage by TACE. Subunits in the EpEX and EpCAM dimers are colored pink and blue, except for the grey transmembrane region. Possible movement of EpEX subunits in the L264C and the destabilized EpCAM dimer is depicted with two positions of the pink subunit and a dashed line with arrows.

Although both mutant proteins have distinct oligomeric states in solution and constitute a useful system for functional studies of EpEX in vitro, their effect in full-length EpCAM remains to be evaluated before the conclusions are applied to EpCAM in a cellular context. Here, the EpCAM transmembrane α-helix is predicted to play a role in dimer stabilization via their intramembrane dimerization (Pavšič et al., 2014). Therefore, the full-length protein with TYloop^mut^ mutations could thus still exist as a dimer, associated via the transmembrane regions. Even if this is indeed the case, we speculate that the abolished interactions between EpEXs would still lead to a less stable dimer that should be more prone to dissociation and consequently to proteolytic processing. On the other hand, it is unlikely EpCAM would get cleaved if subunits remain connected via the transmembrane α-helices, even if the interactions between the ectodomains are abrogated. The interaction between transmembrane regions should confine the movement of EpEX similarly to the disulfide bond in L264C, which is located at its C-terminus (Fig. 4).

## Conclusions

To conclude, we have designed, purified and characterized oligomer-state specific mutants of EpCAM’s ectodomain. With them, we have shown that the destabilization of EpCAM dimer increases its susceptibility towards cleavage by TACE and that the cleavage of a dimeric EpCAM is negligible. We believe that these mutants of EpCAM ectodomain will provide a valuable tool for elucidating the role of the EpCAM oligomeric state either in solution or on the cell surface. Illuminating the role of the individual oligomeric state in interaction with other proteins and how oligomerization itself is regulated will importantly contribute to the understanding of the underlying mechanisms of the biological function of EpCAM.

## Supplemental Information

10.7717/peerj.11484/supp-1 Supplemental Information 1 Effect of mutations on solvatation free energy gain upon formation of the dimer interaction interface. The three individual mutants with the highest change compared to wildtype EpEX are bolded.

10.7717/peerj.11484/supp-2 Supplemental Information 2 Raw data of network edges used to generate the map in Figure 1 B. Network edges represent the frequency of inter-subunit inter-residue contact formation during the MD simulation of EpEX (PDB: 4MZV).

10.7717/peerj.11484/supp-3 Supplemental Information 3 Key inter-subunit inter-residue distances during the MD simulation of EpEX. The table contains distances (in Å) vs time (in ns) during the initial 0.5 ns of the MD simulation.

10.7717/peerj.11484/supp-4 Supplemental Information 4 Key inter-subunit inter-residue distances during the MD simulation of TYloop^mut^. The table contains distances (in Å) vs time (in ns) during the initial 0.5 ns of the MD simulation.

10.7717/peerj.11484/supp-5 Supplemental Information 5 Raw data of Molecular weights analyses. The table contains calculated molecular weights and other characteristics of the main peak in each analyzed sample and BSA standards obtained by OMNISEC RESOLVE+REVEAL system (Malvern Panalytical, UK).

10.7717/peerj.11484/supp-6 Supplemental Information 6 Raw SDS-PAGE gel of Figure 2D. The gel was stained with Coomassie Blue. The sample order is the same as in Figure 2D.

10.7717/peerj.11484/supp-7 Supplemental Information 7 Raw Western blot for Figure 3A - 1. Image shows the signal obtained with ChemiDoc MP Imaging system with default settings for AlexaFluor 647 dye. The sample order is the same as in Figure 3A. This is the first of the three replicates analyzed.

10.7717/peerj.11484/supp-8 Supplemental Information 8 Raw Western blot for Figure 3A - 2. Image shows the signal obtained with ChemiDoc MP Imaging system with default settings for AlexaFluor 647 dye. The sample order is the same as in Figure 3A. This is the second of the three replicates analyzed.

10.7717/peerj.11484/supp-9 Supplemental Information 9 Raw Western blot for Figure 3A - 3. Image shows the signal obtained with ChemiDoc MP Imaging system with default settings for AlexaFluor 647 dye. The sample order is the same as in Figure 3A. This is the third of the three replicates analyzed.

10.7717/peerj.11484/supp-10 Supplemental Information 10 Raw SDS-PAGE gel of Figure 3D. The gel was stained with Coomassie Blue. Sample order is the same as in Figure 3D.

10.7717/peerj.11484/supp-11 Supplemental Information 11 Band intensities obtained from proteolytic cleavage experiments. The table contains raw data used in Figure 3 B and C. The scaling and normalisation calculations are also included.

## Additional Information and Declarations

### Data Availability

The following information was supplied regarding data availability: The solvation-free energy gains for dimerization of wild-type and mutant proteins, calculated with PISA and the raw gels and blots, distances obtained from MD simulations and outputs from protein MW analyses are available in the Supplemental Files.
