# Supplementary material for: Destabilization of EpCAM dimer is associated with increased susceptibility towards cleavage by TACE
Source: PeerJ. 2021 May 21;9:e11484. doi: 10.7717/peerj.11484 (PMC8142927; doi:10.7717/peerj.11484)
Supplement: Supplemental Information 1 — The three individual mutants with the highest change compared to wildtype EpEX are bolded. [file peerj-09-11484-s001.docx]

| protein/mutant | ΔG_interface formation_ [kcal/mol] |
| --- | --- |
| EpEX | -11.6 |
| R80D | -8.4 |
| R81D | -7.5 |
| A82D | -11.6 |
| **K83D** | **-2.6** |
| **P84D** | **-3.5** |
| E85D | -11.6 |
| G86D | -11.9 |
| A87D | -11.2 |
| **L88D** | **-1.7** |
| Q89D | -11.1 |
| N90D | -5.2 |
| K83D, P84D, L88D | 2.6 |
