# Supplementary figures and images for: Destabilization of EpCAM dimer is associated with increased susceptibility towards cleavage by TACE
Source: PeerJ. 2021 May 21;9:e11484. doi: 10.7717/peerj.11484 (PMC8142927; doi:10.7717/peerj.11484)

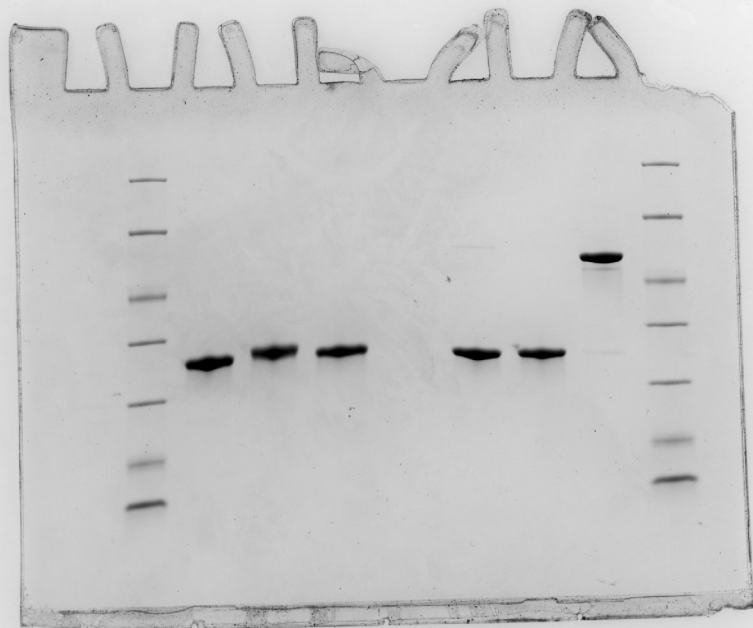

Supplement: Supplemental Information 6 — The gel was stained with Coomassie Blue. The sample order is the same as in Figure 2D. [file peerj-09-11484-s006.pdf]

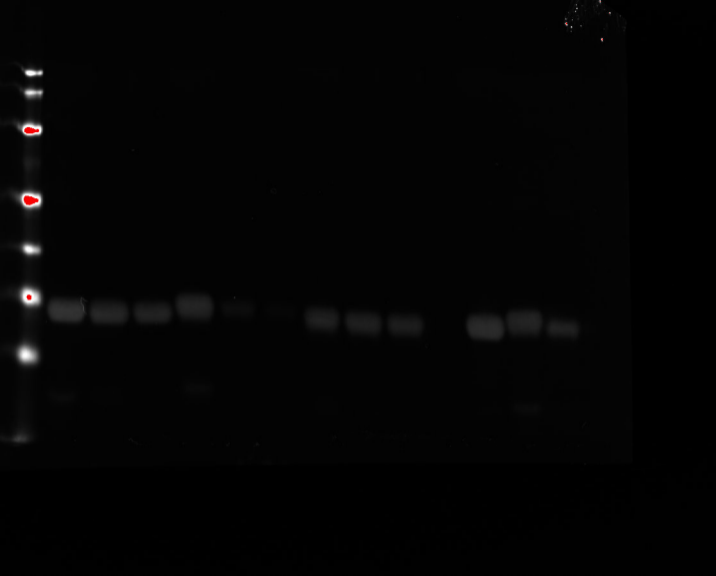

Supplement: Supplemental Information 7 — Image shows the signal obtained with ChemiDoc MP Imaging system with default settings for AlexaFluor 647 dye. The sample order is the same as in Figure 3A. This is the first of the three replicates analyzed. [file peerj-09-11484-s007.pdf]

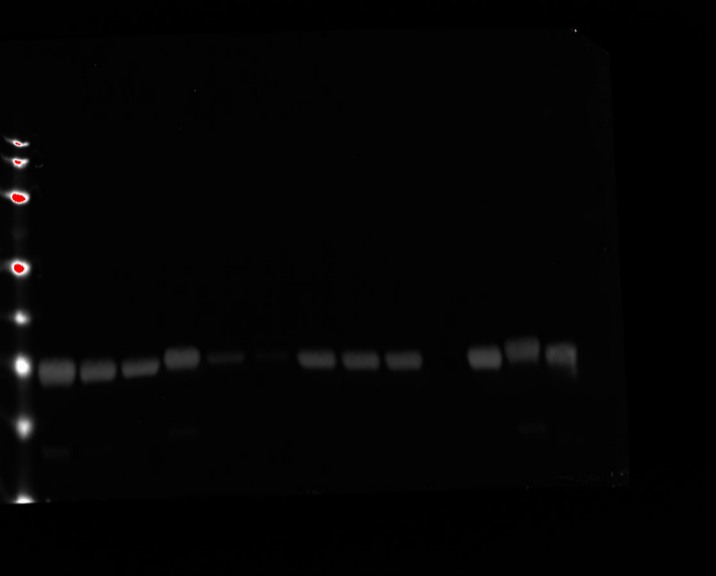

Supplement: Supplemental Information 8 — Image shows the signal obtained with ChemiDoc MP Imaging system with default settings for AlexaFluor 647 dye. The sample order is the same as in Figure 3A. This is the second of the three replicates analyzed. [file peerj-09-11484-s008.pdf]

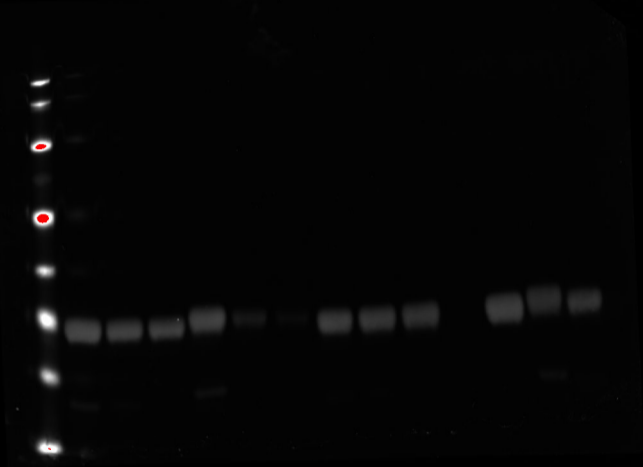

Supplement: Supplemental Information 9 — Image shows the signal obtained with ChemiDoc MP Imaging system with default settings for AlexaFluor 647 dye. The sample order is the same as in Figure 3A. This is the third of the three replicates analyzed. [file peerj-09-11484-s009.pdf]

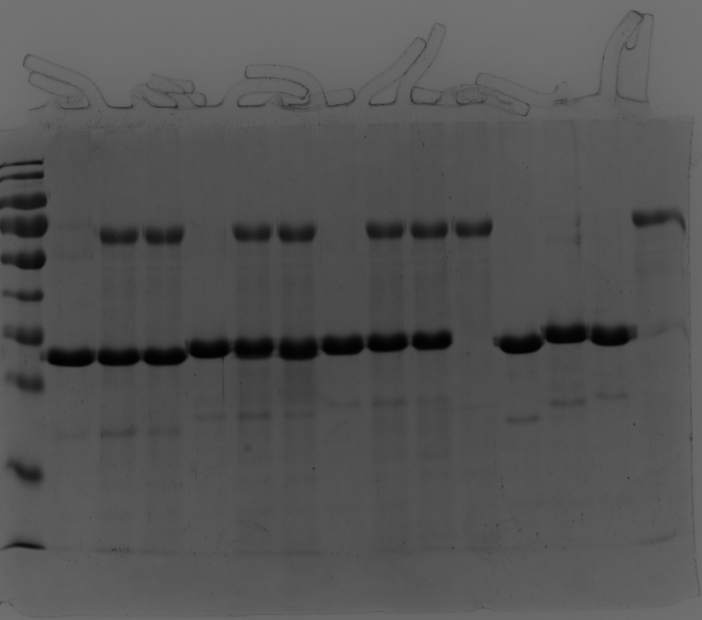

Supplement: Supplemental Information 10 — The gel was stained with Coomassie Blue. Sample order is the same as in Figure 3D. [file peerj-09-11484-s010.pdf]
